# Comparing the protective effects of resveratrol, curcumin and sulforaphane against LPS/IFN-γ-mediated inflammation in doxorubicin-treated macrophages

**DOI:** 10.1038/s41598-020-80804-1

**Published:** 2021-01-12

**Authors:** Haidy A. Saleh, Eman Ramdan, Mohey M. Elmazar, Hassan M. E. Azzazy, Anwar Abdelnaser

**Affiliations:** 1 Department of Chemistry, School of Sciences and Engineering, The American University in Cairo, AUC Avenue, P.O. Box 74, New Cairo, 11835 Egypt; 2 Department of Pharmacology and Toxicology, Faculty of Pharmacy, The British University in Egypt, Cairo, Egypt; 3 Institute of Global Public Health, School of Sciences and Engineering, The American University in Cairo, AUC Avenue, P.O. Box 74, New Cairo, 11835 Egypt

**Keywords:** Chemical genetics, Mechanism of action, Natural products, Chemical biology, Biochemistry, Cytokines, Innate immune cells, Translational immunology

## Abstract

Doxorubicin (DOX) chemotherapy is associated with the release of inflammatory cytokines from macrophages. This has been suggested to be, in part, due to DOX-mediated leakage of endotoxins from gut microflora, which activate Toll-like receptor 4 (TLR4) signaling in macrophages, causing severe inflammation. However, the direct function of DOX on macrophages is still unknown. In the present study, we tested the hypothesis that DOX alone is incapable of stimulating inflammatory response in macrophages. Then, we compared the anti-inflammatory effects of curcumin (CUR), resveratrol (RES) and sulforaphane (SFN) against lipopolysaccharide/interferon-gamma (LPS/IFN-γ)-mediated inflammation in the absence or presence of DOX. For this purpose, RAW 264.7 cells were stimulated with LPS/IFN-γ (10 ng/mL/10 U/mL) in the absence or presence of DOX (0.1 µM). Our results showed that DOX alone is incapable of stimulating an inflammatory response in RAW 264.7 macrophages. Furthermore, after 24 h of incubation with LPS/IFN-γ, a significant increase in tumor necrosis factor-alpha (TNF-α), interleukin-6 (IL-6), and inducible nitric oxide synthase (iNOS) mRNA levels was observed. Similarly, nitric oxide (NO) production and TNF-α and IL-6 protein levels were significantly upregulated. Moreover, in LPS/IFN-γ-treated macrophages, the microRNAs (miRNAs) miR-146a, miR-155, and miR-21 were significantly overexpressed. Interestingly, upon testing CUR, RES, and SFN against LPS/IFN-γ-mediated inflammation, only SFN was able to significantly reverse the LPS/IFN-γ-mediated induction of iNOS, TNF-α and IL-6 and attenuate miR-146a and miR-155 levels. In conclusion, SFN, at the transcriptional and posttranscriptional levels, exhibits potent immunomodulatory action against LPS/IFN-γ-stimulated macrophages, which may indicate SFN as a potential treatment for DOX-associated inflammation.

## Introduction

DOX is the first-choice anthracycline drug used in the treatment of a wide variety of solid tumors and hematological malignancies^1^. The antitumor effect of DOX is mediated through DNA intercalation and topoisomerase 2α (Top2α) isoenzyme inhibition, preventing further DNA synthesis and causing cell death^2^. However, DOX chemotherapy is mainly limited by its cardiotoxicity, which has been recently related to inflammation^3^. For instance, a previous study demonstrated how DOX indirectly activated TLR4 receptors—one of the pattern recognition receptor families that is expressed on macrophages—by disrupting gut flora that then release endotoxins, leading to systemic inflammation and causing damage in several organs^4^. When activated, TLR4 dimerizes and then recruits intracellular adaptor proteins, which in turn activate a sequence of signaling cascades that result in triggering the nuclear translocation of transcription factor nuclear factor-κB (NF-κB), provoking the expression of proinflammatory cytokines, such as TNF-α and IL-6^5^. Notably, macrophages are suggested to be the critical intermediates in inflammation-based cardiac damage induced by DOX chemotherapy^6^. Nevertheless, the direct effects of DOX itself on macrophages are still unclear^7^.

Designing an effective strategy to attenuate TLR4-mediated inflammation is important. Currently, scientific directions are, interestingly, focused on phytochemicals—their anti-inflammatory effects and mechanisms of action^8^. Curcumin (CUR), resveratrol (RES), and sulforaphane (SFN), in particular, are among the most promising natural molecules for the prevention and treatment of several chronic inflammatory and autoimmune disorders, such as rheumatic arthritis, ulcerative colitis, cardiovascular diseases and cancers associated with chronic inflammatory disorders^9–11^. Their mechanisms of action were previously reported to be through the modulation of the TLR4 signaling pathway and macrophage polarization and the downregulation of cytokine overexpression^12^. Nonetheless, the differences in their efficacy and anti-inflammatory mechanisms are not yet known^13,14^.

**Figure 1:**
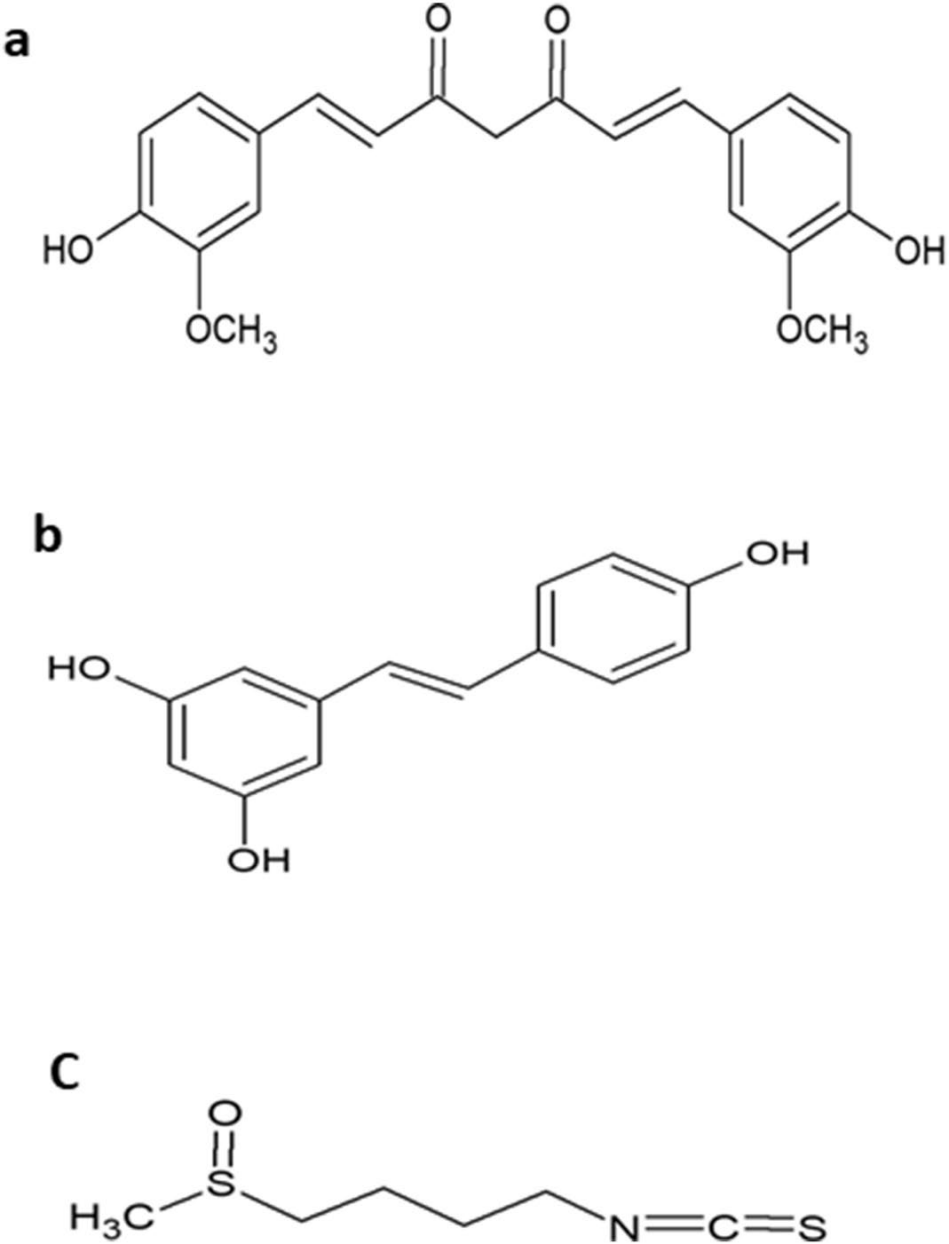
Chemical structure of curcumin (**A**), resveratrol (**B**) and sulforaphane (**C**).

In the present study, we hypothesize that, similar to previously published in vivo findings^4^, DOX by itself does not induce inflammation and that DOX mediates inflammation through a secondary mechanism. Furthermore, we hypothesize that the phytochemicals CUR, RES, and SFN differentially modulate LPS/IFN-γ-mediated inflammation in the absence or presence of DOX. Therefore, the aims of the current study are first to investigate the direct effect of DOX on RAW 264.7 macrophages and determine whether DOX directly stimulates inflammatory signals in these macrophages and, second, to compare the anti-inflammatory effects of the phytochemicals CUR, RES and SFN (Fig. 1) against LPS/IFN-γ-mediated inflammation in macrophages in the absence or presence of DOX.

## Materials and methods

### Materials

LPS (*Escherichia coli* 0111: B4; Cat No. L2630) and curcumin (C_21_H_20_O_6_; ≥ 94%; Cat No. 458-37-7) were purchased from Sigma Chemical Co. (St. Louis, MO, USA). Murine interferon-γ (Cat No. 315-05) was obtained from PeproTech (Rocky Hill, NJ, USA). High-glucose Dulbecco's modified Eagle’s medium (DMEM) was obtained from Gibco (Cat No. 41965-039), fetal bovine serum (FBS) was obtained from Gibco (Cat No. 10270-106), dimethyl sulfoxide (DMSO; Cat No. 67-68-5), chloroform (HPLC grade; Cat No. C607SK-1), isopropanol (HPLC grade; Cat No. BP26324), ethanol (HPLC grade; Cat No. 64-17-5), RevertAid cDNA kit (Cat No. K1621), Maxima SYBR Green qPCR (Cat No. K0251), and mRNA primers (Cat No. 10629186; designed by NCBI primer BLAST tool) were all purchased from Thermo Fisher Scientific (Waltham, MA, USA). A Griess reagent kit (Cat No. G7921) was purchased from Invitrogen (Carlsbad, CA, USA). DMEM (with 4.5 g/L glucose, without L-glutamine and without phenol red) (Cat No. 12-917F), penicillin–streptomycin mixture pen/strep (Cat No. 09-757F), and phosphate-buffered saline (PBS; 10X; Cat No. 17-516Q) were obtained from Lonza Bioscience (Basel, Switzerland). Doxorubicin (Cat No. 25316-40-9) and 3-(4,5-dimethylthiazol-2-yl)-2,5-diphenyltetrazolium bromide (MTT; Cat No. 298-93-1) were purchased from TOCRIS (Bristol, UK). Sulforaphane (C_6_H_11_NOS_2_; ≥ 98%; Cat No. 4478-93-7), resveratrol (C_14_H_12_O_3_; ≥ 98%; Cat No. 501-36-0), murine TNF-α (Cat No. 500850) and IL-6 (Cat No. 583371) enzyme-linked immunosorbent assays **(**ELISAs) were purchased from Cayman Europe OÜ (Tallinn, Estonia). QiAzol lysis buffer (Cat No. 79306), RNAse/DNAse-free water (Cat No. 129114), a miScript II RT kit (Cat No. 218161), a miScript SYBR Green PCR kit (Cat No. 218073), and the following miScript primer assays were purchased from Qiagen (Hilden, Germany): Mm_miR-155_1 (Cat No. MS00001701), Mm_miR-21_2 (Cat No. MS00011487), Mm-miR-146a*_1 (Cat No. MS00024220), and Hs_RNU6-2_11 (Cat No. MS00033740).

### Cell culture

The RAW 264.7 cell line (ATCC TIB-71; RRID: CVCL_0493) was grown in 75 cm^2^ flasks at 37 °C in a 5% CO_2_ humidified incubator until reaching 80% confluence. As suggested by ATCC, cultured cells were maintained in high-glucose DMEM supplemented with 10% heat inactivated fetal bovine serum (FBS) and 1% Pen-Strep (100 units/mL penicillin and 100 µg/mL streptomycin) at 37 °C in humidified air with 5% CO_2_.

### Chemical treatments

When RAW 264.7 cells (passage no. 6–15) reached a density of 2–3 × 10^6^ cells/mL, they were seeded at a density of 2 × 10^5^ cells/well in 96-well plates and cultured for 2 h (for MTT and Griess) or at a density of 2 × 10^6^ cells/well in 6-well plates (for RNA extraction and ELISA) and then stimulated with new medium containing *E. coli* LPS (10 ng/mL) and murine interferon-γ (10 U/mL) for 24 h with or without herbal treatments. Then, DOX (0.1 µM), CUR, RES, and SFN (5, 10, and 20 µM) were each dissolved in dimethyl sulfoxide (DMSO, final concentration of 0.1%) and added separately with or without LPS/IFN-γ to the RAW 264.7 cells. Cells treated with 0.1% (v/v) DMSO served as the vehicle control.

### Cell viability

RAW 264.7 cells were seeded at a density of 2 × 10^5^ cells/well in a 96-well plate and cultured for 2 h. For optimization, the cells were treated with LPS (10 ng and 100 ng) and IFN-γ (5 and 10 U/mL) separately. Various combinations were used to determine the noncytotoxic concentration necessary for stimulating the RAW 264.7 cells. In another experiment, the cells were treated with increasing concentrations of DOX (0.005, 0.01, 0.05, 0.1, or 0.5 µM). Additionally, increasing concentrations of herbal treatments CUR, RES, and SFN (5, 10, and 20 µM) were tested in the presence or absence of LPS/IFN-γ. Based on the optimization results, RAW 264.7 cells in all subsequent experiments were stimulated with 10 ng/ml LPS plus 10 U/mL IFN-γ plus 0.1 µM DOX and coincubated with CUR, RES, or SFN at concentrations of 5, 10, or 20 μM for 24 h at 37 °C in a humidified incubator with 5% CO_2_. Cell viability was analyzed by MTT colorimetric assay as previously described^15^. After 24 h of incubation, the medium was discarded and replaced with 1 mg/ml MTT dissolved in serum-free DMEM. After 2 h of incubation, the formazan crystals that had formed were dissolved in isopropanol. Then, the absorbance was measured at 540 nm using a Nano SPECTROstar microplate reader (BMG LABTECH, Ortenberg, Germany), and the percentage of viable macrophages relative to the control was calculated.

### Nitrite assay

The nitrite concentration in the culture medium was measured as an indicator of NO production. RAW 264.7 cells were seeded at a density of 2 × 10^5^ cells/well in a 96-well plate and cultured for 2 h, stimulated with 10 ng/ml LPS plus 10 U/mL IFN-γ plus 0.1 µM DOX, and coincubated with CUR, RES, or SFN at concentrations of 5, 10, or 20 μM for 24 h at 37 °C in a humidified incubator with 5% CO_2_. The nitrite assay data were analyzed using the Griess method as described previously^16^. From each well, 150 µL of supernatant medium was collected and diluted with 130 µL of deionized water, and then, 20 µL of Griess reagent was added. The plate was maintained in the dark for 30 min. Then, the absorbance was measured at 550 nm with a Nano SPECTROstar microplate reader. The intensity of the color is directly proportional to the nitrite concentration. The nitrite concentration in each sample was calculated by a standard curve produced using NaNO_2_.

### RNA extraction and qPCR

Raw 264.7 cells were seeded at a density of 2 × 10^6^ cells/well in a 6-well plate and cultured for 2 h. Then, the cells were treated with 5 and 20 µM CUR, RES or SFN in the presence of LPS/IFN-γ (10 ng/10 U/mL) plus DOX (0.1 µM) and incubated for an additional 6 h. After 6 h of incubation with test compounds, total RNA was isolated, reverse transcribed, quantified, and analyzed as previously described^16,17^. cDNA was synthesized using a RevertAid First Strand cDNA Synthesis kit according to the manufacturer's instructions. First-strand cDNA was synthesized from 1.0 µg of total RNA with a RevertAid cDNA synthesis kit to obtain mRNA, while the miScript II RT kit was used to obtain miRNA. Quantitative analysis of specific mRNA (TLR4, TNF-α, IL-6, and iNOS) and miRNA (miR-21, miR-146a, miR-155) expression was performed using real-time PCR with SYBR green PCR master mix on an ABI 7500 real-time PCR system (Applied Biosystems). The primers used in the current study were generated using the online NCBI primer design tool (https://www.ncbi.nlm.nih.gov/tools/primer-blast/) and purchased from Thermo Fisher (Table 1).

**Table 1:** Primers Used for qPCR analyses.

Target gene	Primer sequence
iNOS	**Fw:** 5′-GGAACCTACCAGCTCACTCTGG -3′
	**Rv:** 5′-TGCTGAAACATTTCCTGTGCTGT-3′
TLR4	**Fw:** 5′-TTCAGAACTTCAGTGGCTGG-3′
	**Rv:** 5′-TGTTAGTCCAGAGAAACTTCCTG-3′
TNF-α	**Fw:** 5′-GAACTCCAGGCGGTGCCTAT-3′
	**Rv:** 5′-TGAGAGGGAGGCCATTTGGG-3′
IL-6	**Fw:** 5′-GATGCTACCAAACTGGATATAATCAG-3′
	**Rv:** 5′-CTCTGAAGGACTCTGGCTTTG-3′
GAPDH	**Fw:** 5′-CTTTGTCAAGCTCATTTCCTGG-3′
	**Rv:** 5′-TCTTGCTCAGTGTCCTTGC-3′

The following conditions were used for the amplification reactions of mRNA : 10 min at 95 °C and 40 cycles of 95 °C for 15 s and 60 °C for 1 min. The primers were generated using the online NCBI primer design tool (https://www.ncbi.nlm.nih.gov/tools/primer-blast/) and purchased from Thermo Fisher (Table 1). For miR-146a, miR-155 and miR-21, the amplification reactions were performed as follows: 15 min at 95 °C and 40 cycles of 94 °C for 15 s, 55 °C for 30 s and 70 °C for 34 s. The specific custom-made forward miRNA primers were purchased from Qiagen (refer to the materials section). A universal reverse primer for miRNA quantification was included in the miScript SYBR Green PCR Kit. The fold change of the target genes in the treated cells compared to the untreated cells, normalized by the level of GAPDH (for mRNA) or RNU6 (for miRNA), was determined using the following equation: fold change = 2 − Δ(ΔCt), where ΔCt = Ct (target) − Ct (β-actin) and Δ(ΔCt) = ΔCt (treated) − ΔCt (untreated).

### ELISAs

The proinflammatory cytokines TNF-α and IL-6 are secreted after exposure to an inflammatory stimulus. The secretion levels in cell culture supernatant were measured by commercial ELISA kits according to the manufacturer’s instructions (Cayman Europe, Estonia). RAW 264.7 cells were cultured at a density of 2 × 10^6^ cells/well in 6-well plates for 2 h. Then, the cells were treated with 20 µM CUR, RES, and SFN in combination with LPS/IFN-γ plus DOX. After 24 h of incubation, the supernatant was collected, centrifuged and transferred to new microcentrifuge tubes to measure the levels of TNF-α and IL-6 released into the medium. The absorbance was determined at 450 nm using a microplate reader (BMG LABTECH, Ortenberg, Germany).

### Statistical analysis

The data are presented as the mean ± SE for the indicated number of independently performed experiments. One-way ANOVA with Student–Newman–Keuls (SNK) post hoc test was used to identify the statistical significance between multiple groups. A *P*-value < 0.05 was considered statistically significant. A comparative analysis of results between different experimental groups with respect to their corresponding controls was conducted using SigmaPlot (Version 14.0; Systat Software, Chicago, IL, USA), which was also used to draw representative figures. Furthermore, linear regression was performed for all values obtained from the Griess and ELISAs. The percent increase was calculated by taking the fold difference, which is the ratio of the treatment level to its relative control level, and multiplying it by 100.

## Results

### Time- and concentration-dependent effects of LPS and IFN-γ on RAW 264.7 macrophage viability

**Figure 2:**
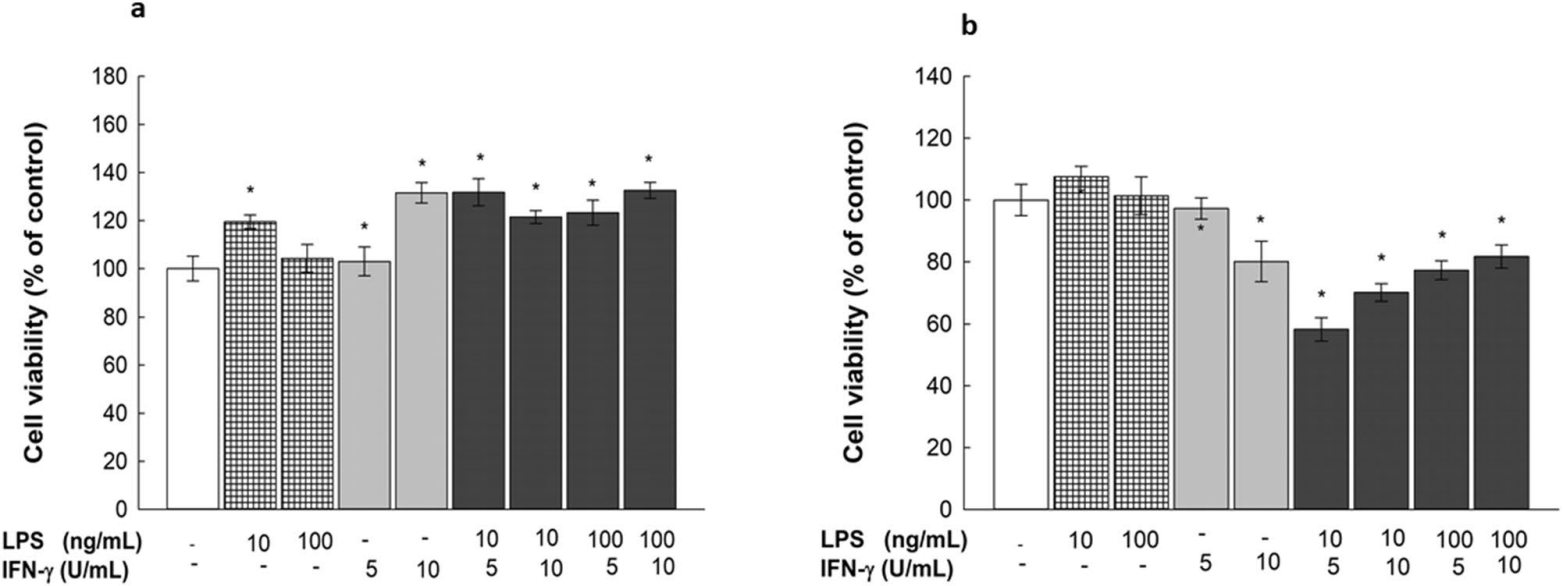
Time- and concentration-dependent effects of LPS and IFN- γ on cell viability in RAW 264.7 macrophages. RAW 264.7 macrophages were exposed to LPS (10 or 100 ng/mL) in the presence or absence of IFN- γ (5 or 10 U/mL) for 24 h (**A**) and 48 h (**B**). Cell cytotoxicity was measured using MTT assay. Data are expressed as a percentage of control (at 100%) ± S.E. (*n* = 8). Comparisons are made with ANOVA followed by Student–Newman–Keuls (SNK) post-hoc test; *, *P* < 0.05, compared with control.

To determine the nontoxic concentrations of LPS and IFN-γ to be used in the present study, RAW 264.7 cells were treated for 24 and 48 h with two concentrations of LPS (10 and 100 ng/mL) in the absence and presence of IFN-γ at 5 and 10 U/mL; thereafter, cytotoxicity was measured by MTT assay. Figure 2A shows no cytotoxicity induced by LPS or IFN-γ, alone or in combination, after 24 h. On the other hand, Fig. 2B shows decreased cell viability after 48 h with some treatments. LPS (10 ng/mL) in the presence of 5 or 10 U/mL IFN-γ decreased cell viability by 42 and 30%, respectively. Similarly, LPS (100 ng/mL) in the presence of 5 or 10 U/mL IFN-γ decreased cell viability by 23 and 19%, respectively. Additionally, individual IFN-γ concentrations (10 U/mL) decreased cell viability by 20%, whereas LPS (10 and 100 ng/mL) and IFN-γ (5 U/mL) did not have a significant effect on cell viability. Based on this information, all subsequent experiments were conducted for 24 h.

### Concentration-dependent effects of LPS and IFN-γ on nitrite production in LPS/IFN-γ-stimulated RAW 264.7 macrophages

**Figure 3:**
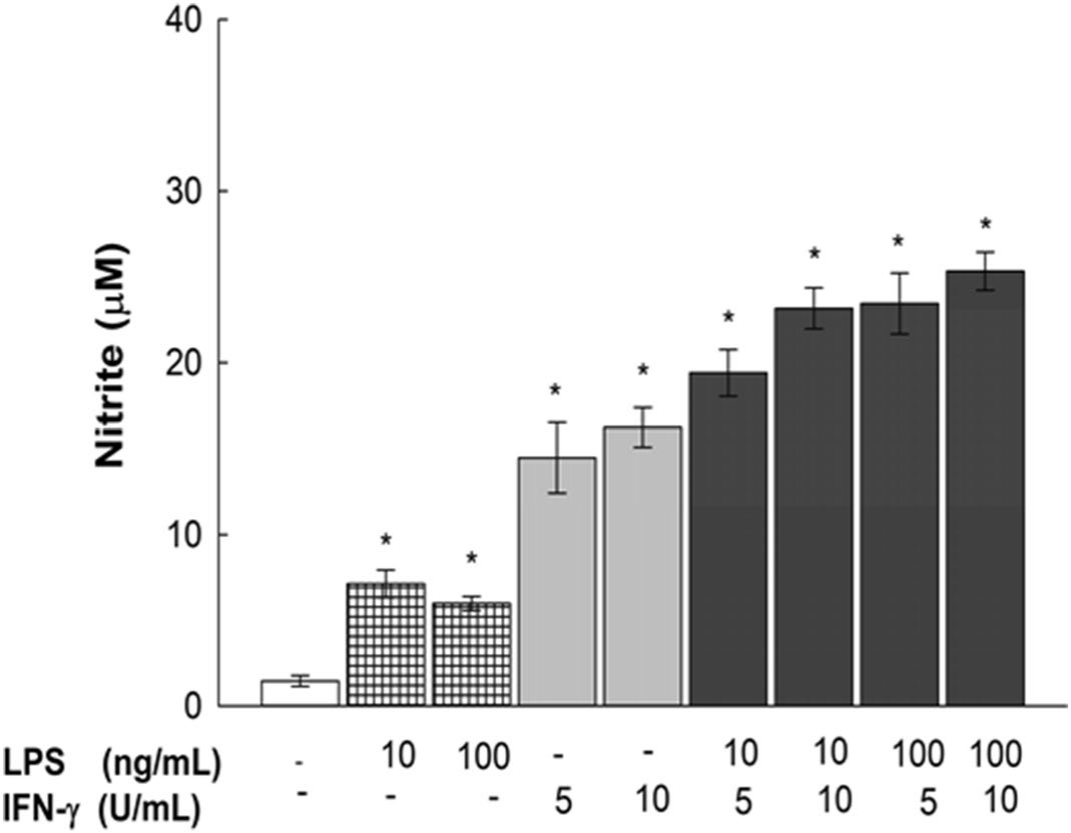
Concentration-dependent effects of LPS and IFN-γ on nitrite production in RAW 264.7 macrophages. RAW 264.7 macrophages were exposed to LPS (10 or 100 ng/mL) in the presence or absence of IFN-γ (5 or 10 U/mL) for 24 h. Data are expressed as mean ± S.E. (*n* = 8). Comparisons are made with ANOVA followed by Student–Newman–Keuls (SNK) post-hoc test; *, *P* < 0.05, compared with control.

To determine the concentrations of LPS and IFN-**γ** to be used for inducing RAW 264.7 cell activation, the individual and combined effects of LPS and IFN-**γ** on RAW 264.7 macrophages were examined. The cells were treated for 24 h with increasing concentrations of LPS (10 and 100 ng/mL) in the absence or presence of IFN-**γ** at 5 and 10 U/mL; thereafter, nitrite production was assessed as an indicator of macrophage activation using the Griess method. Figure 3 shows that exposure to LPS alone at concentrations of 10 and 100 ng/mL induced nitrite production by nearly 500 and 400%, respectively, while exposure IFN-γ at 5 and 10 U/mL alone led to concentration-dependent NO production increases of 1000 and 1150%, respectively. Additionally, different combinations of LPS/IFN-γ showed a seemingly corresponding additive effect with respect to each individual component. Combinations with increasing concentrations showed higher nitrite induction, by 1600%, for both LPS/IFN-γ concentrations of 10 ng/10 U/mL and 100 ng/5 U/mL, whereas the highest tested combination, LPS/IFN-γ at 100 ng/10 U/mL, elevated nitrite by 1700%, which is not significantly different from the effect of LPS/IFN-γ at 10 ng/10 U/mL. Based on this information, all subsequent studies were conducted using 10 ng/mL LPS and 10 U/ml IFN-γ.

### Concentration-dependent effects of DOX on RAW 264.7 macrophages

**Figure 4:**
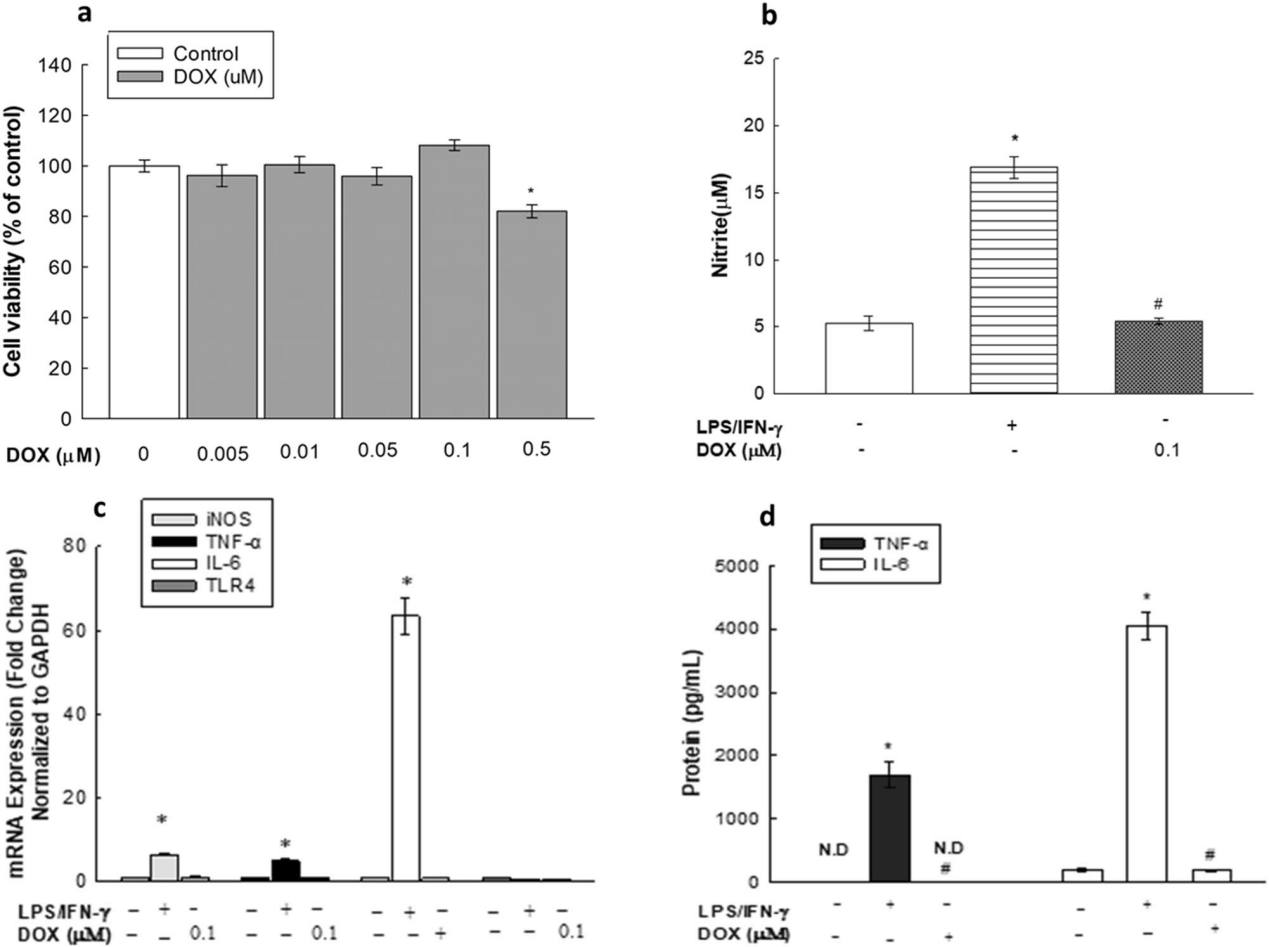
Concentration-dependent effects of DOX on RAW 264.7 macrophages. Cells were exposed to increasing concentrations of DOX (0.005, 0.01, 0.05, 0.1, or 0.5 µM) for 24 h and cell cytotoxicity was measured using MTT assay (**A**). In (**B**, **C** and **D**), cells were exposed to DOX (0.1 µM) or LPS (10 ng/mL) plus IFN-γ (10 U/mL). Nitrite production was determined using Griess method (**B**). mRNA levels of iNOS, TNF-α, IL-6 and TLR4 were measured using qPCR and were normalized to GAPDH (**C**). Protein levels of TNF-α and IL-6 were quantified using ELISA (**D**). Data are expressed as a percentage of control (at 100%) ± S.E. (*n* = 8) for cell viability, or as mean ± S.E. (*n* = 3) for nitrite, qPCR and ELISA. Comparisons are made with ANOVA followed by Student–Newman–Keuls (SNK) post-hoc test; *, *P* < 0.05, compared with control; #, *P* < 0.05, compared with LPS/IFN-γ group.

To examine the direct inflammatory effect of DOX on RAW 264.7 macrophages and to determine whether its inflammatory effect can be exhibited independent of LPS/IFN-γ, RAW 264.7 macrophages were treated with increasing concentrations of DOX (0.005–0.5 µM) to first determine the noncytotoxic concentrations with MTT assays. Figure 4A shows that DOX at concentrations of 0.005–0.1 µM did not significantly affect cellular viability, whereas 0.5 µM DOX decreased cell viability by 18%. Therefore, DOX at a concentration of 0.1 µM was subsequently used to assess the effect of DOX on nitrite levels (Fig. 4B), iNOS, TNF-α, IL-6 and TLR4 mRNA expression levels (Fig. 4C), and TNF-α and IL-6 protein levels (Fig. 4D). As shown in Fig. 4B,C and D, DOX alone did not have any significant effect in comparison to the control (untreated cells) on NO production (Fig. 4B) or iNOS, TNF-α, IL-6 or TLR4 mRNA expression levels (Fig. 4C). Similarly, no significant change was observed in TNF-α and IL-6 protein levels (Fig. 4D) in the DOX-treated RAW 264.7 macrophages. In contrast, the positive control (LPS/IFN-γ) showed an increase in nitrite production of 300% (Fig. 4B) and in the mRNA levels of iNOS, TNF-α and IL-6 of 650%, 500% and 6300%, respectively (Fig. 4C). Last, this stimulatory effect of LPS/IFN-γ on TNF-α and IL-6 mRNA expression was further translated to the respective protein levels (Fig. 4D). Taken together, these results demonstrate that DOX alone is incapable of stimulating RAW 264.7 macrophages; therefore, none of the inducible inflammatory mediators normally associated with activated macrophages were expressed.

### Concentration-dependent effects of CUR, RES and SFN on the viability of RAW 264.7 macrophages

**Figure 5:**
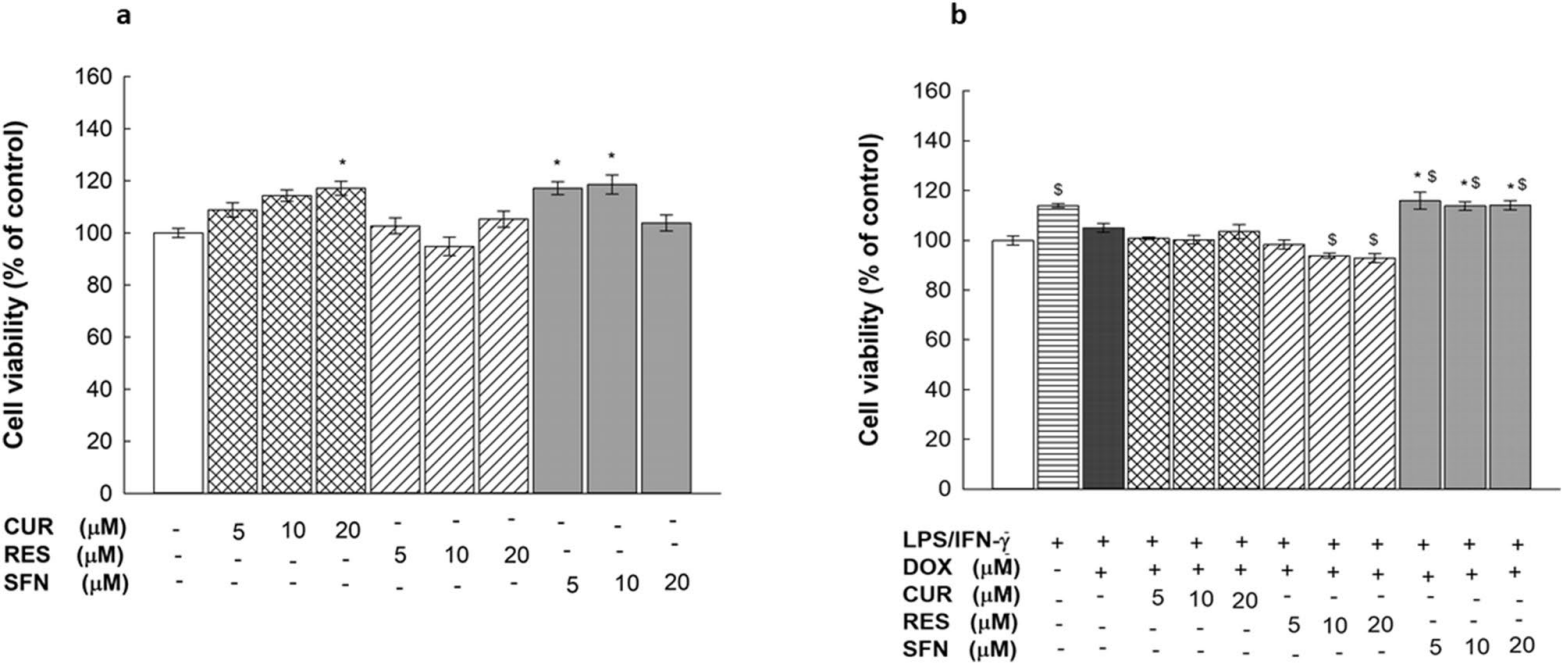
Concentration-dependent effects of CUR, RES and SFN on cell viability in RAW 264.7 macrophages. RAW 264.7 macrophages were exposed for 24 h to CUR, RES or SFN at increasing concentrations of (5, 10 or 20 µM) alone (**A**) or with LPS (10 ng/mL) plus IFN-γ (10 U/mL) in the presence of DOX (0.1 µM) (B). Cell cytotoxicity was measured using MTT assay. Data are expressed as a percentage of control (at 100%) ± S.E. (*n* = 8). Comparisons are made with ANOVA followed by Student–Newman–Keuls (SNK) post-hoc test; *, *P* < 0.05, compared with control; $, *P* < 0.05, compared with LPS/IFN-γ plus DOX group.

To determine the nontoxic concentrations of CUR, RES, and SFN to be used in the present study, RAW 264.7 cells were treated for 24 h with increasing concentrations of treatment compounds (5–20 µM) alone and in combination with LPS/IFN-**γ** (10 ng/10 U/mL) plus DOX (0.1 µM); thereafter, cytotoxicity was measured by MTT assay. Figure 5A shows that exposure to CUR, RES, or SFN separately at 5–20 µM did not significantly affect cell viability. When cells were stimulated with LPS/IFN-**γ** in the presence of DOX, none of the phytochemically treated cells showed significantly affected viability. SFN (10 and 20 µM) conserved its proliferative effect and induced even lower cytotoxicity than the control cells (Fig. 5B).

### Concentration-dependent effects of CUR, RES and SFN on nitrite production in RAW 264.7 macrophages

**Figure 6:**
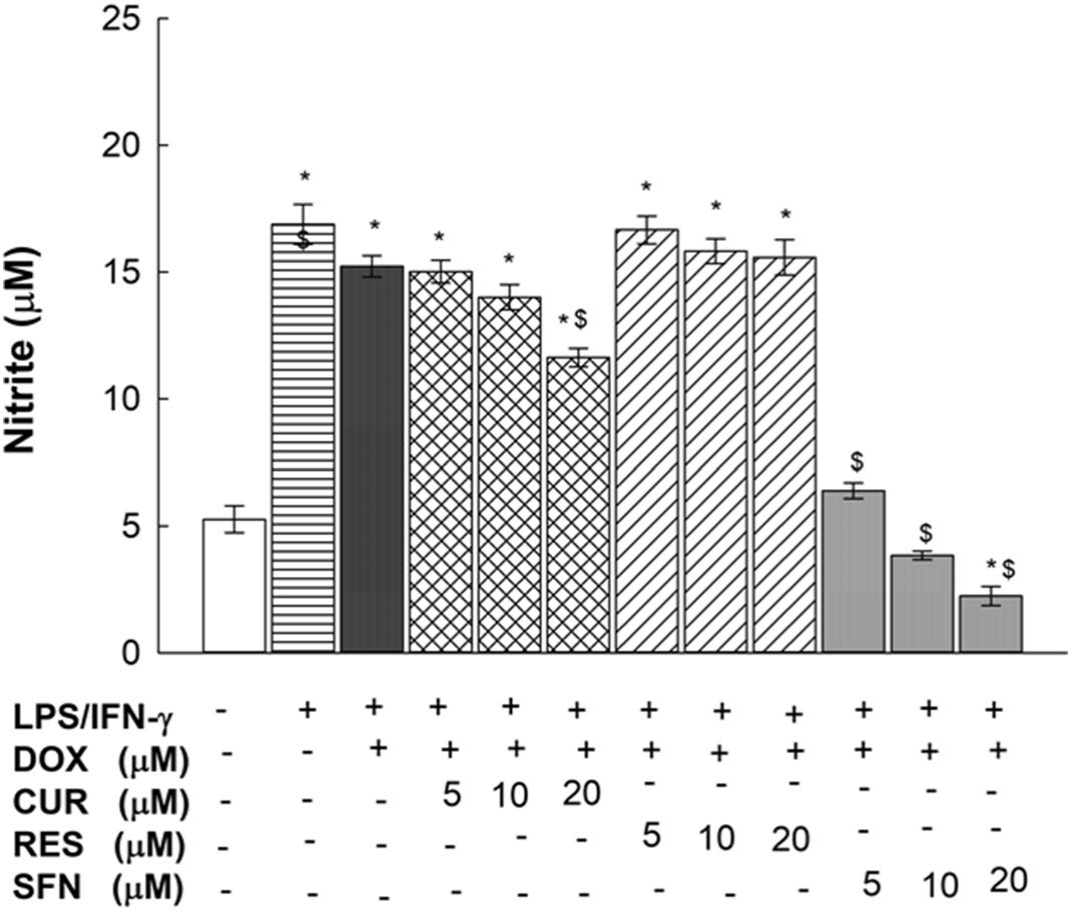
Concentration-dependent effects of CUR, RES and SFN on nitrite production in LPS/IFN- γ-stimulated RAW 264.7 macrophages. RAW 264.7 macrophages were exposed for 24 h to CUR, RES or SFN at increasing concentrations of (5, 10 or 20 µM) in the presence of LPS (10 ng/mL) plus IFN-γ (10 U/mL) with DOX (0.1 µM). Nitrite production was determined using Griess method. Data are expressed as mean ± S.E. (*n* = 8). Comparisons are made with ANOVA followed by Student–Newman–Keuls (SNK) post-hoc test; *, *P* < 0.05, compared with control; $, *P* < 0.05, compared with LPS/IFN-γ plus DOX group.

To examine the effect of the tested phytochemicals on nitrite production, RAW 264.7 cells were exposed for 24 h to increasing concentrations of CUR, RES, or SFN (5—20 µM) in the presence or absence of LPS/IFN-γ (10 ng/10 U/mL) plus DOX (0.1 µM). Thereafter, nitrite levels were assessed using the Griess method. Figure 6 shows that LPS/IFN-γ in the presence of DOX induced nitrite levels by 300%. When cells were coexposed to the phytochemicals and LPS/IFN-γ plus DOX, SFN (5 – 20 µM) inhibited nitrite levels by 58, 75, and 87%, respectively, in a concentration-dependent manner, and CUR at only 20 µM decreased nitrite induction by 23%, whereas RES did not show any significant effect on nitrite production (Fig. 6). Additionally, the effect of SFN in the absence of DOX was assessed, and our results did not show a significant difference in the changed nitrite level percentages between the DOX^**+**^ and DOX^**–**^ treated groups (Supplementary Fig. 2A).

### Effects of CUR, RES and SFN on iNOS mRNA expression levels in LPS/IFN-γ-stimulated RAW 264.7 macrophages

**Figure 7:**
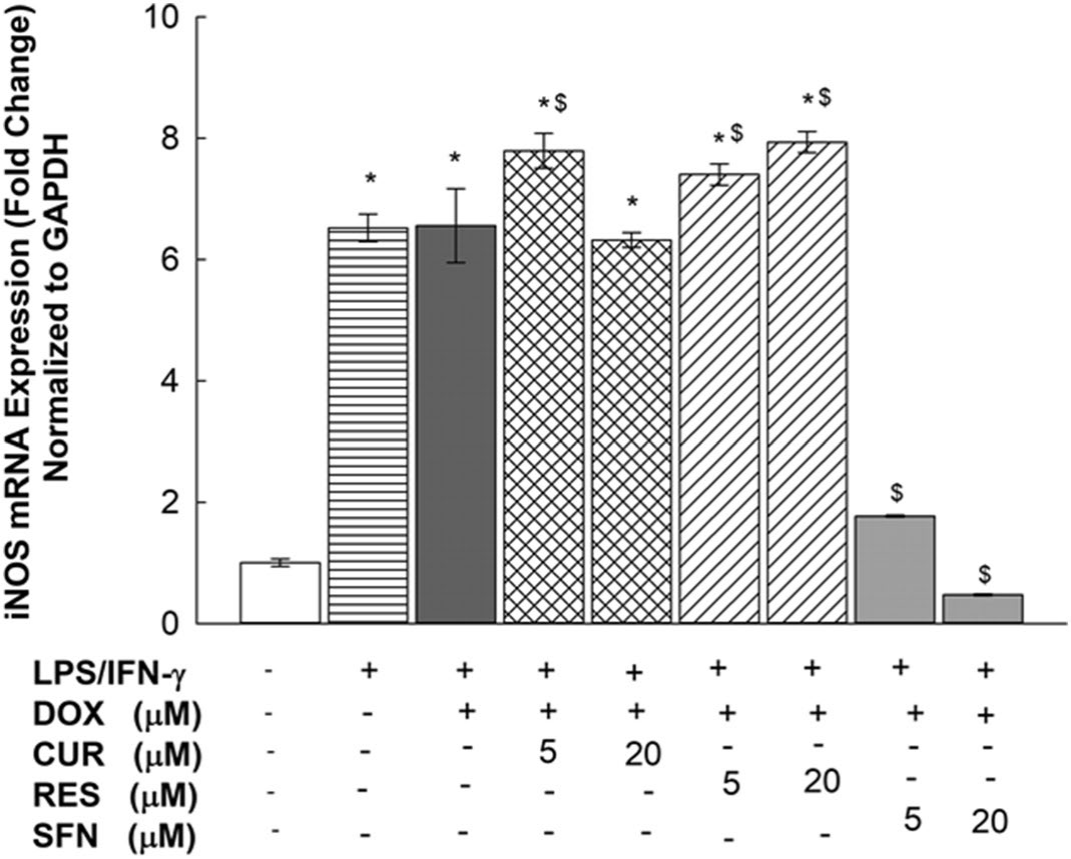
Effects of CUR, RES and SFN on iNOS mRNA expression levels in LPS/IFN- γ-stimulated RAW 264.7 macrophages. RAW 264.7 cells were treated for 6 h with CUR, RES or SFN (5 and 20 M) in the presence of LPS (10 ng/mL) plus IFN-γ (10 U/mL) with DOX (0.1 µM). iNOS mRNA levels were measured using qPCR and were normalized to GAPDH. Data are expressed as mean ± S.E. (*n* = 3). Comparisons are made with ANOVA followed by Student–Newman–Keuls (SNK) post-hoc test; *, *P* < 0.05, compared with control; $, *P* < 0.05, compared with LPS/IFN- γ plus DOX treatment.

To examine whether the inhibitory effect of the treatments on nitrite production was due to interference with iNOS mRNA expression, RAW 264.7 cells were exposed for 6 h to two concentrations of CUR, RES, or SFN (5 and 20 µM) in the presence of LPS/IFN-γ (10 ng/10 U/mL) plus DOX (0.1 µM). Thereafter, iNOS mRNA levels were measured using real-time PCR. As shown in Fig. 7, LPS/IFN-γ induced iNOS mRNA by 650%. However, when these cells were cotreated with LPS/IFN-γ plus DOX in the presence of the tested phytochemicals, SFN (5 and 20 µM) downregulated iNOS mRNA levels by 74 and 94%, respectively, whereas CUR and RES did not show any significant inhibitory effect at any of the tested concentrations (Fig. 7). In the absence of DOX, SFN at all tested concentrations induced similar changes to iNOS levels with no significant differences in the percentages of change (Supplementary Fig. 2B).

### Effects of CUR, RES and SFN on TLR4 mRNA expression levels in LPS/IFN-γ-stimulated RAW 264.7 macrophages

**Figure 8:**
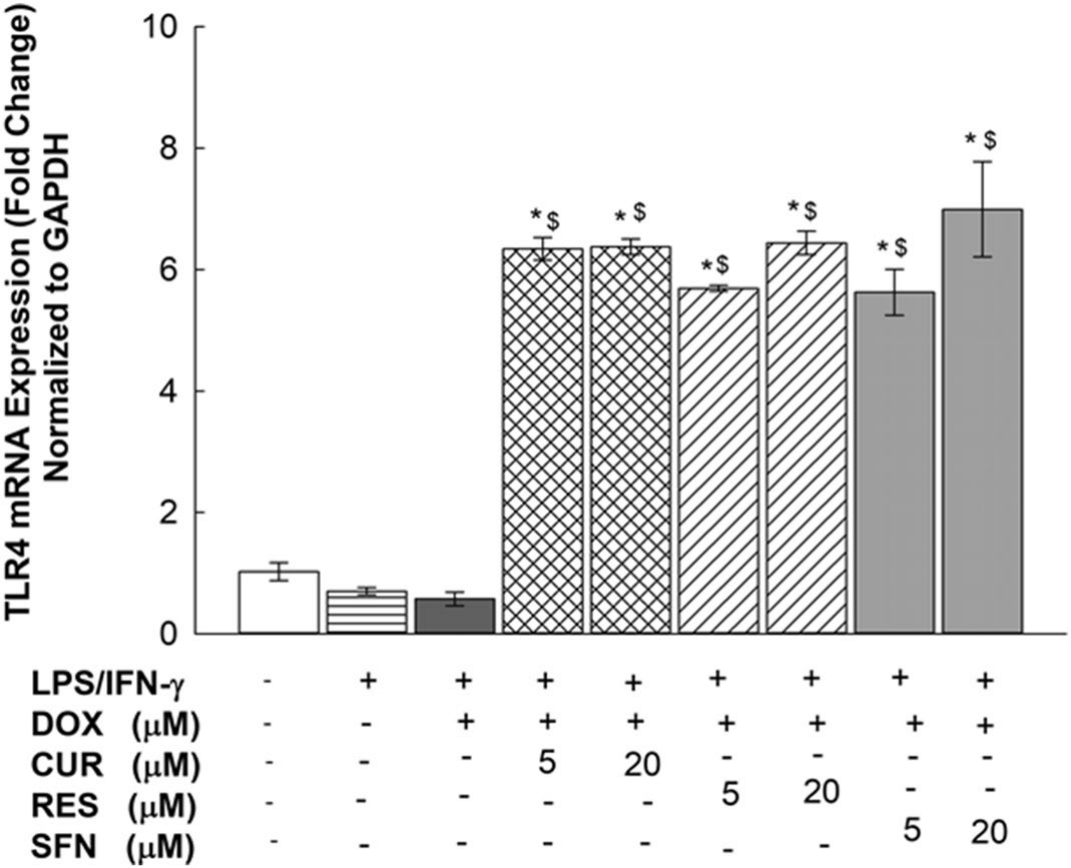
Effects of CUR, RES and SFN on TLR4 mRNA expression levels in LPS/IFN-γ-stimulated RAW 264.7 macrophages. Cells were treated for 6 h with CUR, RES or SFN (5 and 20 µM) in the presence of LPS (10 ng/mL) plus IFN-γ (10 U/mL) with DOX (0.1 µM). TLR4 mRNA levels were measured using qPCR and were normalized to GAPDH. Data are expressed as mean ± S.E. (*n* = 3). Comparisons are made with ANOVA followed by Student–Newman–Keuls (SNK) post-hoc test; *, *P* < 0.05, compared with control; $, *P* < 0.05, compared with LPS/IFN- γ plus DOX treatment.

To examine the effect of the target treatments on TLR4 mRNA levels, RAW 264.7 cells were exposed for 6 h to two concentrations of CUR, RES, or SFN (5 and 20 µM) in the presence or absence of LPS/IFN-γ (10 ng/10 U/mL) plus DOX (0.1 µM). Thereafter, TLR4 mRNA levels were measured using real-time PCR. As shown in Fig. 8, LPS/IFN-γ downregulated TLR4 mRNA levels by 50%, a difference that was not significant. When cells were coexposed to LPS/IFN-γ plus DOX in the presence of CUR, RES or SFN, the TLR4 mRNA levels were increased by approximately 630% with CUR (5 and 20 µM), 570 and 640% with RES (5 µM and 20 µM), respectively, and 560 and 700% with SFN (5 and 20 µM), respectively (Fig. 8). Additionally, the effect of SFN in the absence of DOX was assessed, and our results did not show a significant difference in the percent changes of TLR4 between the DOX^+^ and DOX^–^ treated groups (Supplementary Fig. 2B).

### Effects of CUR, RES and SFN on TNF-α and IL-6 mRNA expression levels in LPS/IFN-γ-stimulated RAW 264.7 macrophages

**Figure 9:**
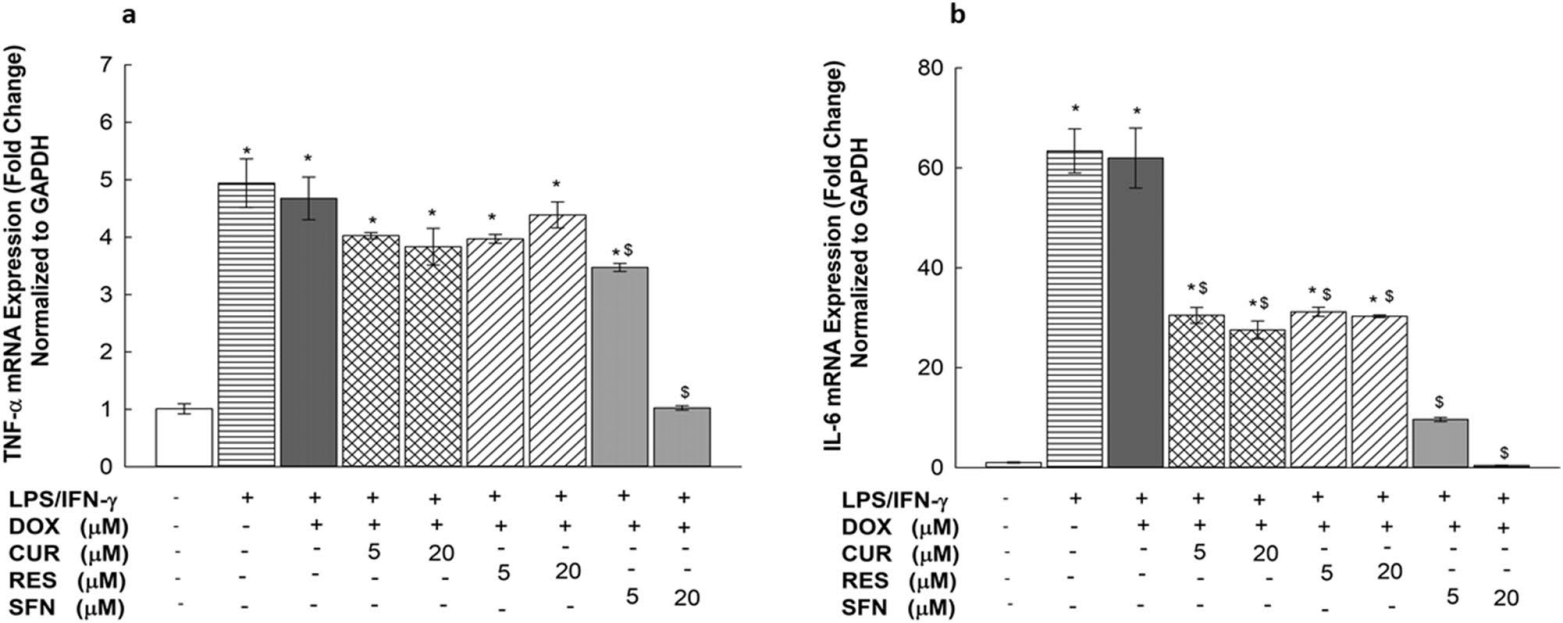
Effects of CUR, RES and SFN on TNF-α and IL-6 mRNA expression levels in LPS/IFN- γ-stimulated RAW 264.7 macrophages. Cells were treated for 6 h with CUR, RES or SFN (5 and 20 M) in the presence of LPS (10 ng/mL) plus IFN-γ (10 U/mL) with DOX (0.1 µM). TNF-α (**A**) and IL-6 (**B**) mRNA levels were measured using qPCR and were normalized to GAPDH. Data are expressed as mean ± S.E. (*n* = 3). Comparisons are made with ANOVA followed by Student–Newman–Keuls (SNK) post-hoc test; *, *P* < 0.05, compared with control; $, *P* < 0.05, compared with LPS/IFN- γ plus DOX treatment.

To examine the effect of the experimental treatments on TNF-α and IL-6 mRNA levels, RAW 264.7 cells were exposed for 6 h to two concentrations of CUR, RES, or SFN (5 and 20 µM) in the presence of LPS/IFN-γ (10 ng/10 U/mL) with or without DOX (0.1 µM). Thereafter, TNF-α and IL-6 mRNA levels were measured by real-time PCR. As shown in Fig. 9, LPS/IFN-γ in the presence and absence of DOX significantly induced IL-6 and TNF-α expression, by 6300 and 500%, respectively. When cells were cotreated with the tested phytochemicals, SFN (5 and 20 µM) significantly attenuated LPS/IFN-γ-induced mRNA levels of TNF-α by 26 and 78%, respectively, and IL-6 attenuated these levels by 84 and 100%, respectively. On the other hand, CUR and RES did not show any significant inhibition of TNF-α-induced mRNA levels at any tested concentration, but both downregulated the IL-6 mRNA expression levels in the induced macrophages by approximately 50% at 5 and 20 µM (Fig. 9). Similarly, SFN was studied with LPS/IFN-γ-induced macrophages without DOX, and the results showed approximately the same percent changes in TNF-α and IL-6 levels (Supplementary Fig. 2B).

### Effects of CUR, RES and SFN on TNF-α and IL-6 protein levels in LPS/IFN-γ-stimulated RAW 264.7 macrophages

**Figure 10:**
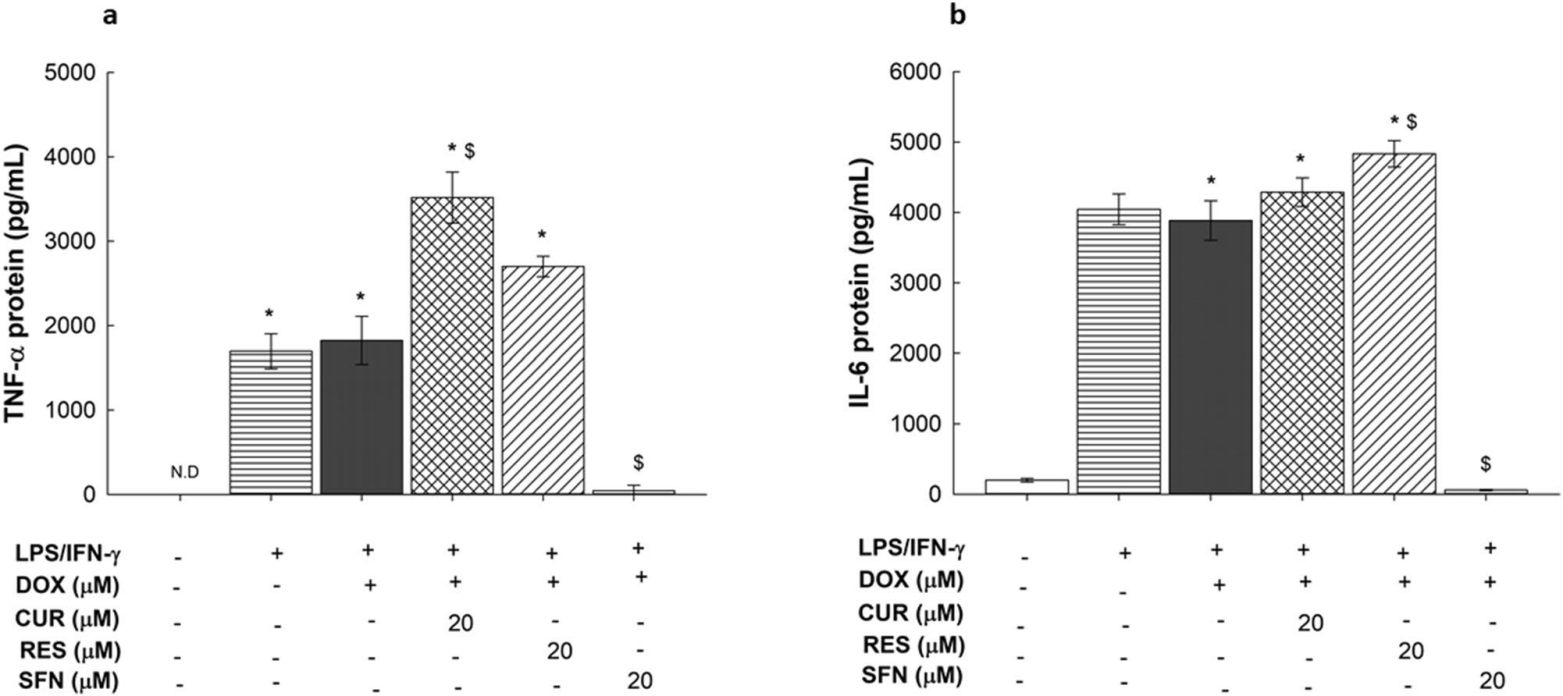
Effects of CUR, RES and SFN on TNF-α and IL-6 protein levels in LPS/IFN- γ-stimulated RAW 264.7 macrophages. Cells were treated for 24 h with CUR, RES or SFN (5 and 20 M) in the presence of LPS (10 ng/mL) plus IFN-γ (10 U/mL) with DOX (0.1 µM). TNF-α (A) and IL-6 (B) protein levels were quantified using ELISA. Data are expressed as mean ± S.E. (*n* = 3). Comparisons are made with ANOVA followed by Student–Newman–Keuls (SNK) post-hoc test; *, *P* < 0.05, compared with control; $, *P* < 0.05, compared with LPS/IFN- γ plus DOX treatment.

To determine whether the SFN-mediated inhibition of LPS/IFN-γ-induced increases in TNF-α and IL-6 mRNA levels, compared to the effect of CUR and RES, translated to increased protein levels, RAW 264.7 cells were exposed to 20 µM CUR, RES, and SFN for 24 h in the presence of LPS/IFN-γ (10 ng/10 U/mL) plus DOX (0.1 µM). Thereafter, the protein expression levels of TNF-α and IL-6 were assessed using ELISAs. Figure 10A shows that LPS/IFN-γ in the presence or absence of DOX increased TNF-α levels to almost 1750 pg/mL compared with the control (TNF-α levels were undetected in untreated control cells). On the other hand, IL-6 protein levels were induced to almost 2000% of the control (Fig. 10B). When the cells were cotreated with the tested phytochemicals in the presence of LPS/IFN-γ and DOX, SFN downregulated both TNF-α- and IL-6-induced expression by almost 98%, whereas CUR and RES did not show any inhibitory effect on either IL-6- or TNF-α-induced expression levels (Fig. 10). Similar results were observed in LPS/IFN-γ-induced cells not treated with DOX (Supplementary Fig. 2C).

### Basal and LPS/IFN-γ-induced relative expression of miR-146a, miR-155 and miR-21 miRNAs in RAW 264.7 macrophages

**Figure 11:**
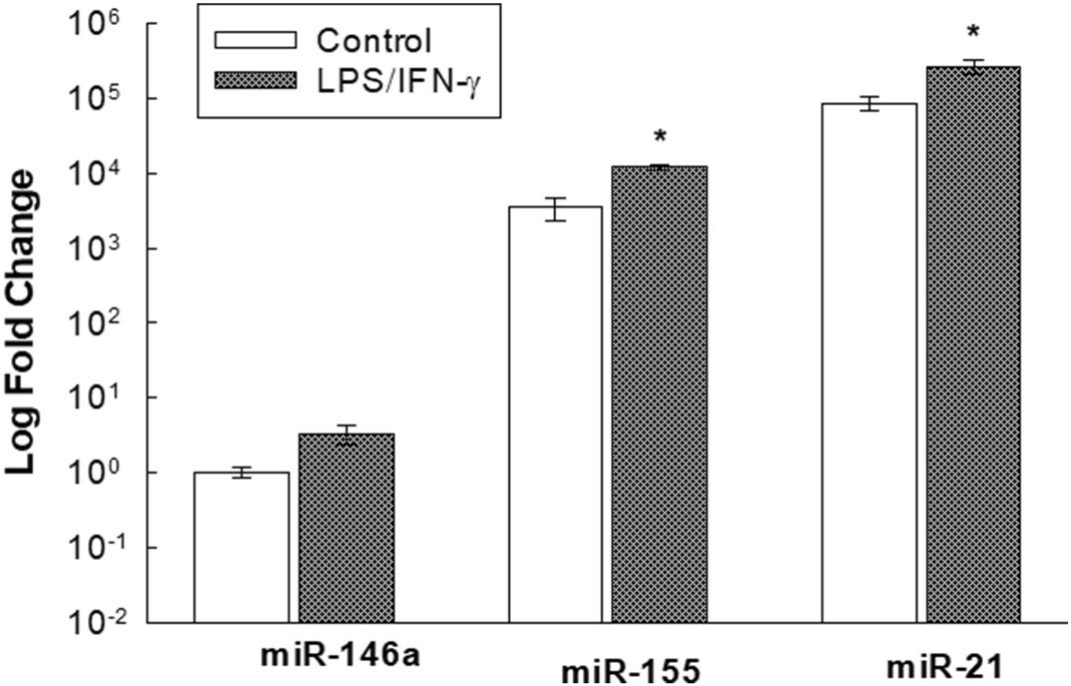
Basal and LPS/IFN-γ-induced relative expression of miR-146a, miR-155 and miR-21 miRNAs in RAW 264.7 macrophages. Cells were treated for 6 h with LPS (10 ng/mL) plus IFN-γ (10 U/mL). The basal and induced expression levels of miR-146a, miR-155, and miR-21 were measured using qPCR and were normalized to RNU6. Data are expressed as mean ± S.E. (*n* = 3). Comparisons are made with ANOVA followed by Student–Newman–Keuls (SNK) post-hoc test; *, *P* < 0.05, compared with corresponding control.

A novel comparison was conducted on the relative expression levels of the tested miRNAs in RAW 264.7 macrophages using real-time PCR. The results data are presented as log_10_ fold changes. As shown in Fig. 11, at the basal level without LPS/IFN*-*γ stimulation, miR-146a was expressed at the lowest level among miRNAs in the RAW 264.7 cells, and miR-21 was expressed at a higher level than miR-146a, with a 5-log greater fold change. On the other hand, miR-155 expression was higher than miR-146a expression, with a 3.5-log greater log change. Upon LPS/IFN*-*γ (10 ng/10 U/mL) stimulation, miR-146a was induced to increase by only a 0.5-log fold change, whereas both miR-155 and miR-21 were increased significantly, by a 1-log fold change, relative to their respective controls, which corresponded to their basal expression relative to miR-146a (Fig. 11).

### Effects of SFN on miR-146a, miR-155 and miR-21 miRNA expression levels in LPS/IFN-γ-stimulated RAW 264.7 macrophages

**Figure 12:**
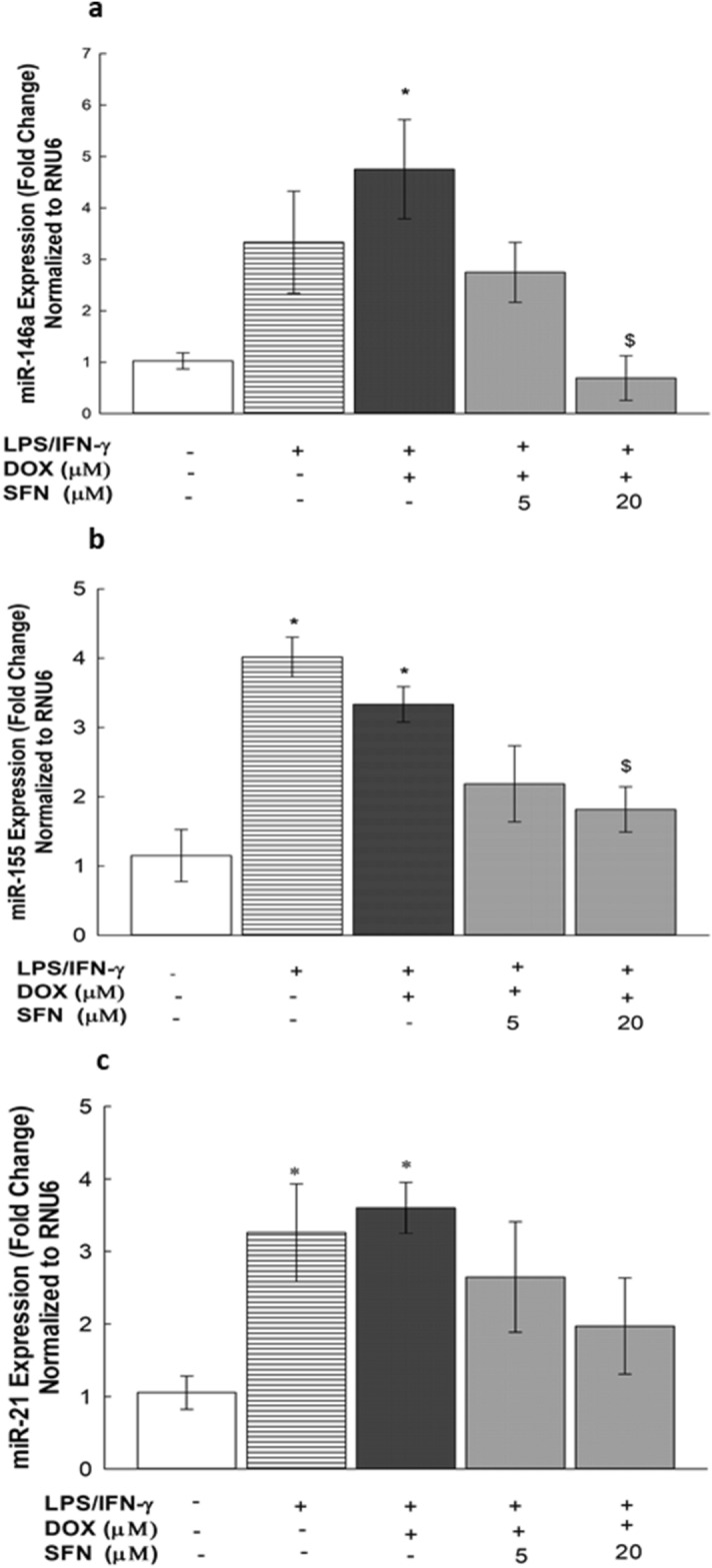
Effects of SFN on miR-146a, miR-155 and miR-21 miRNAs expression levels in LPS/IFN- γ-stimulated RAW 264.7 macrophages. Cells were treated for 6 h with SFN (5 and 20 µM) in the presence of LPS (10 ng/mL) plus IFN-γ (10 U/mL) with DOX (0.1 µM. The expression levels of miR-146a (**a**), miR-155 (**b**), and miR-21 (**c**) were measured using qPCR and were normalized to RNU6. Data are expressed as mean ± S.E. (*n* = 3). Comparisons are made with ANOVA followed by Student–Newman–Keuls (SNK) post-hoc test; *, *P* < 0.05, compared with control; $, *P* < 0.05, compared with LPS/IFN-γ plus DOX treatment.

To determine whether the SFN-mediated effect on the LPS/IFN-γ-induced stimulation of different inflammatory markers is mediated through an epigenetic mechanism, we examined the potential effect of SFN on miR-146a, miR-155, and miR-21 expression using real-time PCR. For this purpose, RAW 264.7 cells were exposed to SFN (5 and 20 µM) in the presence of LPS/IFN-γ (10 ng/10 U/mL) plus DOX (0.1 µM) for 24 h. Our results showed that miR-146a, miR-155 and miR-21 were upregulated by almost 500, 300 and 350%, respectively (Fig. 12A,B, and C). On the other hand, when cells were cotreated with SFN (5 and 20 µM), the induced miR-146a and miR-155 levels were significantly attenuated by 85 and 40%, respectively, with 20 µM SFN (Fig. 12A,B), whereas no significant effect on the miR-21 level was observed (Fig. 12C).

## Discussion

In one prominently proposed mechanism, DOX mediates its inflammatory response through LPS-induced systemic inflammation^4^. In the present study, we investigated the direct and indirect effects of DOX on the activation of RAW 264.7 macrophages through the TLR4 signaling pathway. Gene expression analyses of proinflammatory mediators, namely, iNOS, TNF-α, and IL-6, revealed a significant increase in these mediators after LPS/IFN-γ stimulation in the presence or absence of DOX, whereas no significant change was detected with DOX treatment alone. These data exclude the possibility that DOX has a direct effect on TLR4 signaling in RAW 264.7 macrophages. Our second main finding was that SFN, in comparison to CUR and RES, effectively attenuated RAW 264.7 macrophage LPS/IFN-γ stimulation by antagonizing TLR4 signaling in macrophages, as evidenced by significantly suppressed expression of iNOS, TNF-α, and IL-6 at both the transcriptional and translational levels. Interestingly, we also found that SFN, at the epigenetic level, significantly downregulated LPS/IFN-γ-induced expression of miR-155 and miR-146a, which are key regulatory miRNAs of the TLR4-mediated inflammatory response.

DOX has been previously suggested to induce systemic inflammation by promoting intestinal disruption in mice, causing leakage of endotoxins into the circulation, which bind to TLR4 receptors on macrophages, activating the NF-κB pathway and subsequently inducing inflammatory cytokine generation^4,14,18^. Nonetheless, almost no data have been provided on the exact mechanism by which DOX affects macrophages^7^. In the current study, our data showed that there was no significant direct or additive effect of DOX on RAW 264.7 macrophages when used alone or in combination with LPS/IFN-γ at different concentrations (Fig. 4 and Supplementary Fig. 1). Pertaining to the absence of the intestinal epithelium factor in vitro, the observed inability of DOX to directly stimulate murine macrophages comports with the hypothesized mechanism previously presented^4^. Although DOX did not show a direct positive effect on macrophages, we cotreated macrophages with LPS/IFN-γ and DOX in an attempt to simulate the in vivo study conditions and to rule out any interference of DOX on the pharmacological activity of the tested phytochemicals. In an attempt to significantly activate macrophages without inducing any cytotoxicity, we determined the levels of nitrite production with different concentrations of LPS and IFN-γ individually and in combination (Fig. 3), and our data showed a synergistic effect of LPS and IFN- γ in inducing a significant inflammatory response in RAW 264.7 macrophages, which is consistent with the findings of Reis et al.^19^ and accepted mechanisms for macrophage activation and polarization^20^. Additionally, a negative correlation between LPS/IFN-γ incubation time and cell viability was observed (Fig. 2), which is in line with the report of Shi et al.^21^. Considering this finding, we incubated cells with LPS/IFN-γ for only 24 h in all subsequent experiments to confirm that the stimulatory effect of LPS and IFN-γ is independent of induced cytotoxicity. Moreover, a recent report demonstrated the role of IFN-γ in inducing the rapid activation of aerobic glycolysis, which sustains the viability and proinflammatory activity of M1 macrophages^22^. In parallel, MAPK activation by LPS is known to stimulate macrophage proliferation^23^. This evidence supports the preservative effect of LPS/IFN-γ observed in RAW 264.7 cells (Fig. 2A), which agrees with the findings of Fresta et al. showing no cytotoxic effect of LPS/IFN-γ on RAW 264.7 cells^24^.

In an attempt to identify the molecular mechanisms of the proinflammatory effect of LPS/IFN-γ on RAW 264.7 macrophages, we assessed the gene expression of different inflammatory markers mediated by the TLR4 pathway. From these analyses, we observed a significant upregulation of iNOS mRNA levels and nitrite production (Figs. 6 and 7), as confirmed by Fresta et al.^24^. In addition, the remarkable overexpression of IL-6 and TNF-α at both the mRNA and protein levels in the LPS/IFN-γ-treated RAW 264.7 macrophages (Figs. 9 and 10) affirmed the cause-effect relationship between LPS/TLR4 signal transduction and proinflammatory cytokine release from macrophages^5^. Since macrophage sensitivity to LPS and IFN-γ is partly regulated at the level of the TLR4 receptor^5^, we assessed the expression level of TLR4 in LPS/IFN-γ-stimulated macrophages (Fig. 8). Although TLR4 upregulation was reported in LPS-stimulated human monocytic cells (THP-1) upon LPS stimulation^25^, our findings showed no significant fold change in TLR4 expression levels upon LPS/IFN-γ stimulation of RAW 264.7 macrophages (Fig. 8). Similar to our data, growing evidence is indicating that TLR4 mRNA expression levels remained constant in rat alveolar macrophages and human monocytes after LPS exposure^26,27^, which can be explained as a negative regulatory feedback mechanism that enhance macrophage tolerance toward further LPS stimulation^28^.

Indeed, devising a strategy to counteract TLR4-mediated inflammation is of great importance to overcome DOX limitations. Recent reports have indicated that 66% of therapeutic natural plants have outstanding anti-inflammatory and antioxidant potential^29^. CUR, RES, and SFN are most commonly known for their antagonistic effect against TLR4 signaling in macrophages^30,31^. They act as mitigating agents against several chronic inflammatory and autoimmune disorders^9–11^. Therefore, they are considered promising agents for conferring protection against LPS/IFN-γ-mediated inflammation involved in DOX chemotherapy. Interestingly, our results revealed that all tested phytochemicals upregulated TLR4 mRNA expression in LPS/IFN-γ-activated and inactivated macrophages (Fig. 8). This effect likely indicates direct antagonistic effect of the tested phytochemicals on the TLR4 receptor, which was previously mentioned in several studies^30,31^. This evidence further supports a possible refractory upregulation of the receptor in response to the antagonistic activity of the experimental phytochemicals.

In addition, our data offer the first points of comparison between the protective effects of the target phytochemicals on LPS/IFN-γ-induced RAW 264.7 macrophages, revealing that SFN, in comparison to CUR and RES, exhibited significant dose-dependent inhibition of LPS/IFN-γ-induced proinflammatory markers in DOX-treated RAW 264.7 cells. For example, SFN significantly downregulated TNF-α and IL-6 cytokines (Figs. 9 and 10), NO production and enzyme critical for its generation, iNOS (Figs. 6 and 7). NO is an important defense molecule against infection; however, the excessive production of NO induces cytotoxicity^32,33^. Additionally, SFN exhibited a significant proliferative effect on murine RAW 264.7 macrophages at all tested concentrations (Fig. 5), which may be explained by the study of Shih et al. that showed a promoting effect of SFN on immune cells, as evidenced by T and B cell proliferation upon SFN treatment^34^. On the other hand, RES and CUR showed a noticeable inhibitory effect only on IL-6 mRNA expression, and this effect failed to extend to the protein level (Fig. 9B and 10B). Collectively, our findings indicate an antagonistic effect of SFN that is in agreement with the data published by Ruhee et al. showing that SFN suppressed the release of NO, TNF-α, and IL-6 in LPS-primed RAW 264.7 macrophages^35^.

Taken together, our data raised the possibility that the weak activity of CUR and RES, in comparison to SFN, might be due to a modest efficacy or a dose–effect relationship property of CUR and RES that limited their effects, especially when used at relatively low concentrations; notably, the CUR and RES concentrations (5—20 µM) used in our study were based on our cell viability data (Fig. 5). As illustrated by Chen et al., weak inhibition of TNF-α and IL-1β was reported in RAW 264.7 cells exposed to low concentrations (5 and 10 µM) of CUR^36^. Similarly, a study by Matsuguchi et al. highlighted the necessity of using high concentrations of CUR (50 µM or higher) to obtain a significant inhibitory effect on TLR signaling^37^. Another report by Nelson and his colleagues criticized the instability, lack of potency and selective target activity of CUR^30,38^. Similarly, only a high concentration of RES, 60 µM, was able to induce TLR4 inhibition in LPS-induced RAW 264.7 cells, as indicated by Yang et al.^39^. Given the extensive evidence obtained thus far, our results present a novel comparative observation indicating that SFN shows better pharmacological efficacy than CUR and RES and thus may be a promising antagonist of the TLR4-dependent pathway in DOX-treated murine macrophages. This effect was shown to be independent of DOX interference (Supplementary Fig. 2).

Recent reports have demonstrated an intriguing relationship between the TLR4 pathway and target miRNAs, especially miR-146a, miR-155 and miR-21, in macrophages^40^. These miRNAs, which are short noncoding RNAs, are known to regulate key biological processes via the suppression of gene expression at posttranscriptional levels^41^. They are not only upregulated downstream of TLR4/NF-κB axis activation but also play key regulatory roles in the TLR4 signaling pathway^42^. Our results present, for the first time, a novel comparison between the relative expression levels of the target miRNAs, miR-146a, miR-155, and miR-21, in RAW 264.7 macrophages, showing relatively higher expression of miR-155 and miR-21 than miR-146a in the basal and LPS/IFN-γ-induced states of macrophages (Fig. 11). Although the role of miR-146a in LPS-induced RAW 264.7 macrophage cells remains unclear, our results showed a significant upregulation of miR-146a in LPS/IFN-γ-primed macrophages, which comport with the results of Yong et al., who indicated increased levels of miR-146a in LPS-treated macrophages^41^. Notably, miR-146a is known to downregulate NF-κB-downstream inflammatory mediators and target TLR4 pathway components, such as TRAF6 (tumor necrosis factor receptor-associated family) and IRAK (interleukin-1 receptor-associated kinase), which activate downstream NF-κB and inflammatory cytokines^43–45^. Moreover, as shown in Fig. 11, the significant upregulation of miR-155 after LPS/IFN-γ stimulation is in agreement with the findings of Bala and his colleagues, showing enhanced miR-155 levels upon LPS stimulation of RAW 264.7 cells^46^. Previous reports showed that miR-155 exerts a positive feedback effect on NF-κB signaling by either increasing TNF-α half-life and translation^46^ or suppressing the two negative regulators of TLR4-induced inflammation, suppressor of cytokine signaling 1 (SOCS1) and SH2 (Src homology 2)-containing inositol phosphatase-1 (SHIP-1), enhancing MAPK activity and further stimulating inflammatory cytokine release from macrophages^42,47,48^. This information correlates with the observed upregulation of TNF-α and IL-6 in LPS/IFN-γ-induced RAW 264.7 macrophages (Figs. 9 and 10). In contrast to miR-155, miR-21 is known as a negative regulator of the TLR4 pathway that inhibits NF-κB activation and enhances the expression of anti-inflammatory cytokine IL-10 through the inhibition of programmed cell death protein 4 (PDCD4), which is an IL-10 inhibitor^49,50^. Our data showed that miR-21 was overexpressed upon LPS/IFN-γ stimulation (Fig. 11), which comports with previous studies, presenting an upregulation of miR-21 levels in LPS-treated RAW 264.7 macrophages^51,52^. In the present study, we demonstrated the effect of miR-21 expression in LPS/IFN-γ-stimulated macrophages. However, the role of miR-21 in different types of macrophages is controversial and further investigation is needed^53^.

Inflammatory miRNA modulation is now becoming a novel therapeutic approach^54^. Based on these observations, we hypothesized that the anti-inflammatory effect of SFN can be extended to modulate the overexpression of these target miRNAs in LPS/IFN-γ-stimulated RAW 264.7 macrophages. In line with the study of Deramaudt et al.^55^, our findings revealed that SFN significantly downregulated miR-146a levels (Fig. 12A), which might be explained as dependent-downregulatory negative feedback in response to the SFN inhibitory effect on TLR4 signaling^56^. Similarly, in Fig. 12B, the concentration-dependent downregulation of miR-155 by SFN is consistent with the reports of Wagner et al. and Eren et al. illustrating a concentration-related inhibition of miR-155 by SFN in LPS-treated RAW 264.7 cells and N9 microglial cells, respectively^57,58^. From this evidence, it is tempting to speculate that miR-155 and NF-κB signaling pathway components engage in cross talk, suggesting a need for further investigation in the presence of SFN and other anti-inflammatory phytochemicals^57,59^. In addition, we excluded a role for miR-21 in a possible mechanism for the antagonistic effect of SFN on the TLR4 pathway, since SFN showed no significant effect on miR-21-induced expression in LPS/IFN-γ-activated RAW 264.7 macrophages, novel data presented for the first time (Fig. 12C). A recent study by Cho and his colleagues showed that SFN ameliorated LPS-mediated PDCD4 mRNA reduction in RAW 264.7 cells, but this group could not reveal the underlying mechanism behind this finding and speculated that it is related to miR-21 overexpression^60^. In contrast to their speculation, our findings provide a clue suggesting that miR-21 plays a negligible role in PDCD4 suppression in RAW 264.7 macrophages, which still needs further verification because it may be more significant at higher concentrations of SFN. In this context, the present study suggests a novel posttranscriptional mechanism underlying the effectiveness of SFN as an anti-inflammatory agent due to its multiple sites of action in the TLR4 signaling pathway and highlights a new therapeutic approach that targets TLR4-responsive inflammatory miRNAs, which may help mitigate LPS/TLR4-mediated inflammation, which is known to be involved in DOX treatment.

## Conclusion and future direction

Our results ruled out any direct activity of DOX on RAW 64.7 macrophages and showed that SFN, in comparison to CUR and RES, successfully modulates TLR4 signaling activation in RAW 264.7 macrophages by effectively inhibiting LPS/IFN-γ-induced NO, iNOS, TNF-α, and IL-6 gene expression. Next, our findings introduced the epigenetically inhibitory role of SFN on the expression of miR-155 and miR-146, which implies a regulatory relationship with the TLR4/NF-κB signaling pathway. Because evidence for cross talk between these target miRNAs and TLR4 signaling in the presence of phytochemicals is unclear, future mechanistic investigations utilizing in vitro and in vivo models are needed. Moreover, future experiments should focus on enhancing the delivery of phytochemicals, for example, by either designing novel nanostructures or using different combinations of phytochemicals that work synergistically. Altogether, our data provide evidence for the anti-inflammatory potential of SFN at the transcriptional and posttranscriptional levels and thus indicate that SFN is an effective immunomodulatory agent against LPS/IFN-γ-mediated inflammation, which usually limits the use of DOX chemotherapy.

## Supplementary Information

Supplementary Information 1.

Supplementary Information 2.

Supplementary Information 3.
