# Supplementary figures and images for: Comparing the protective effects of resveratrol, curcumin and sulforaphane against LPS/IFN-γ-mediated inflammation in doxorubicin-treated macrophages
Source: Sci Rep. 2021 Jan 12;11:545. doi: 10.1038/s41598-020-80804-1 (PMC7803961; doi:10.1038/s41598-020-80804-1)

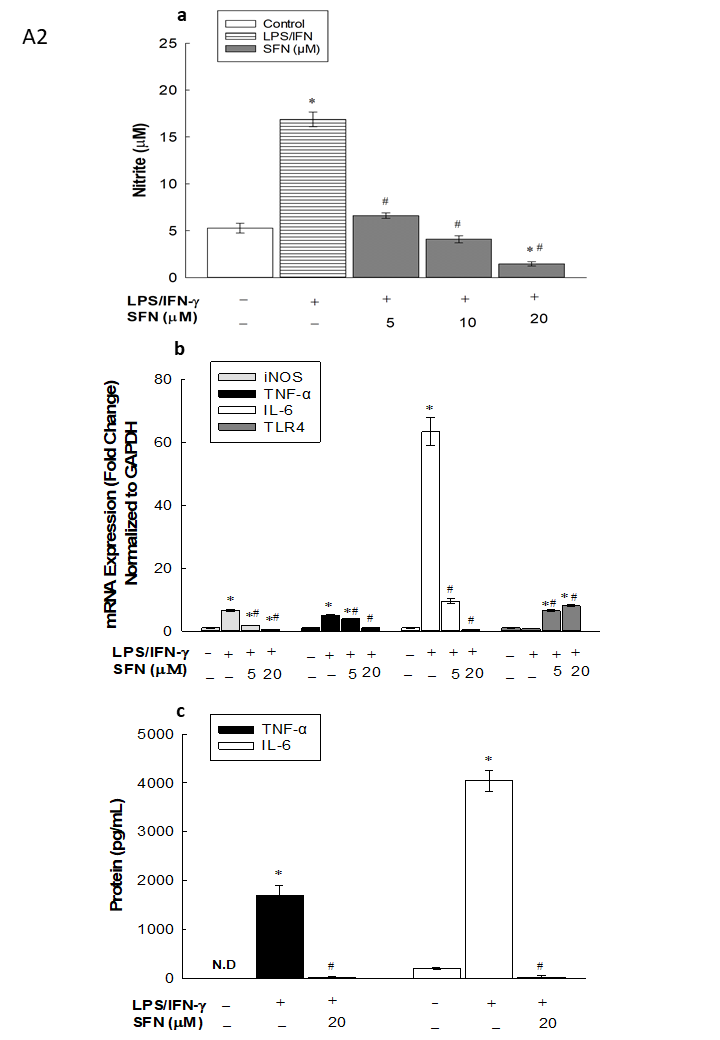

Supplement: Supplementary file 1 — Supplementary Information 1. [file 41598_2020_80804_MOESM1_ESM.tif]

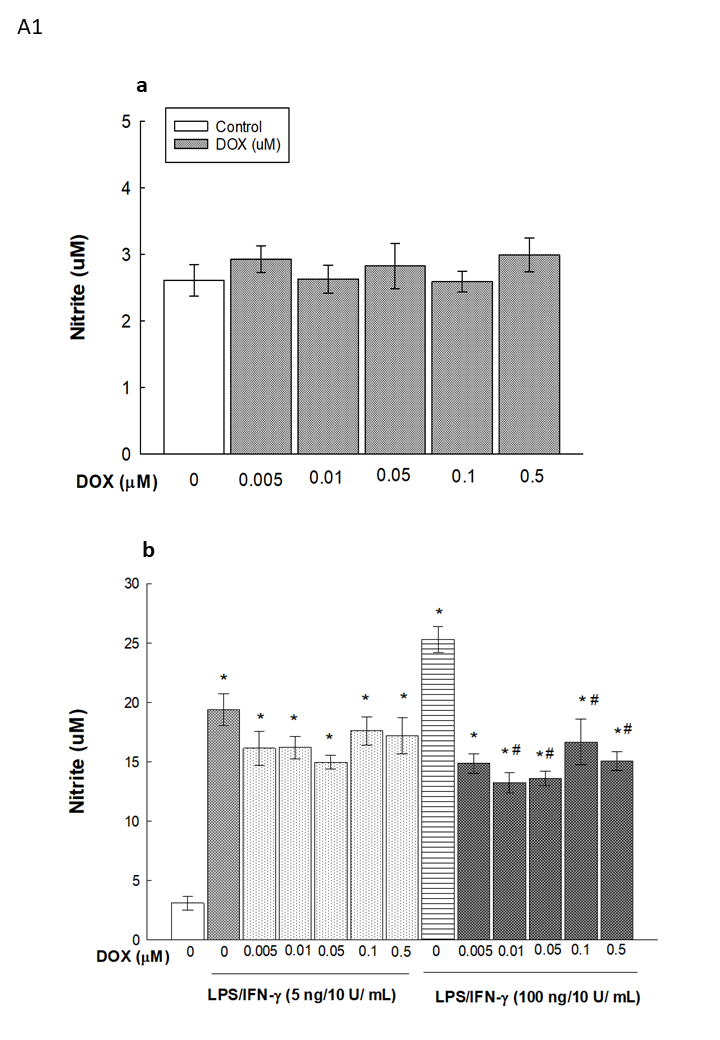

Supplement: Supplementary file 2 — Supplementary Information 2. [file 41598_2020_80804_MOESM2_ESM.tif]
