# Supplementary material for: Comparing the protective effects of resveratrol, curcumin and sulforaphane against LPS/IFN-γ-mediated inflammation in doxorubicin-treated macrophages
Source: Sci Rep. 2021 Jan 12;11:545. doi: 10.1038/s41598-020-80804-1 (PMC7803961; doi:10.1038/s41598-020-80804-1)
Supplement: Supplementary file 3 — Supplementary Information 3. [file 41598_2020_80804_MOESM3_ESM.docx]

**APPENDIX**

**App. Fig. 1** Concentration-dependent effects of DOX in the presence and absence of LPS/IFN-γ in RAW 264.7 macrophages. (A) RAW 264.7 macrophages were exposed to increasing concentrations of DOX (0.005, 0.01, 0.05, 0.1, or 0.5 µM) for 24 h; thereafter nitrite production was determined using Griess method. In (B), cells were co-exposed to increasing concentrations of DOX (0.005, 0.01, 0.05, 0.1, or 0.5 µM) with LPS (10 or 100 ng/mL) plus IFN-γ (5 or 10 U/mL). Data are expressed as mean ± S.E. (*n=*8). Comparisons are made with ANOVA followed by Student–Newman–Keuls (SNK) post-hoc test; *, *P* <0.05, compared with control; #, *P* <0.05, compared with LPS/IFN-γ group.

**App. Fig. 2** Effect of SFN on LPS/IFN-γ-activated RAW 264.7 macrophages. Cells are exposed to LPS (10 ng/mL) plus IFN-γ (10 U/mL) in the presence of SFN (5, 10 and 20 µM) for 24 h, for nitrite level determination using Griess method (A), or for 6 h with SFN (10 and 20 µM) for mRNA levels of iNOS, TNF-α, IL-6 and TLR4 *via* qPCR (B), or for 24 h with SFN (20 µM) for protein levels quantification of TNF-α and IL-6 using ELISA (C). Data are expressed as mean ± S.E. (*n=*3). Comparisons are made with ANOVA followed by Student–Newman–Keuls (SNK) post-hoc test; *, *P* <0.05, compared with control; #, *P* <0.05, compared with LPS/IFN-γ group.
